# Supplementary material for: An ex vivo system to study cellular dynamics underlying mouse peri-implantation development
Source: Dev Cell. 2022 Feb 7;57(3):373–386.e9. doi: 10.1016/j.devcel.2021.12.023 (PMC8826647; doi:10.1016/j.devcel.2021.12.023)
Supplement: Document S1. Figures S1–S5 and Tables S1 [file mmc1.pdf]

Developmental Cell, Volume *57*

Supplemental information

An *ex vivo* system to study cellular dynamics underlying mouse peri-implantation development

Takafumi Ichikawa, Hui Ting Zhang, Laura Panavaite, Anna Erzberger, Dimitri Fabrèges, Rene Snajder, Adrian Wolny, Ekaterina Korotkevich, Nobuko Tsuchida-Straeten, Lars Hufnagel, Anna Kreshuk, and Takashi Hiiragi

Supplemental Figures

Figure S1

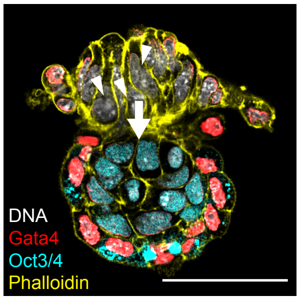

Figure S1. Embryos undergoing ExE invagination, Related to Figure 1

Immunofluorescence of a representative E4.75 embryo in which pTE cells invaginate and form ExE, stained for Oct3/4^{+} EPI, Gata4^{+} VE, actin and DNA. White arrowheads indicate the invaginating pTE cells, while a white arrow indicates the direction of invagination.

Scale bar, 50 μm.

Figure S2

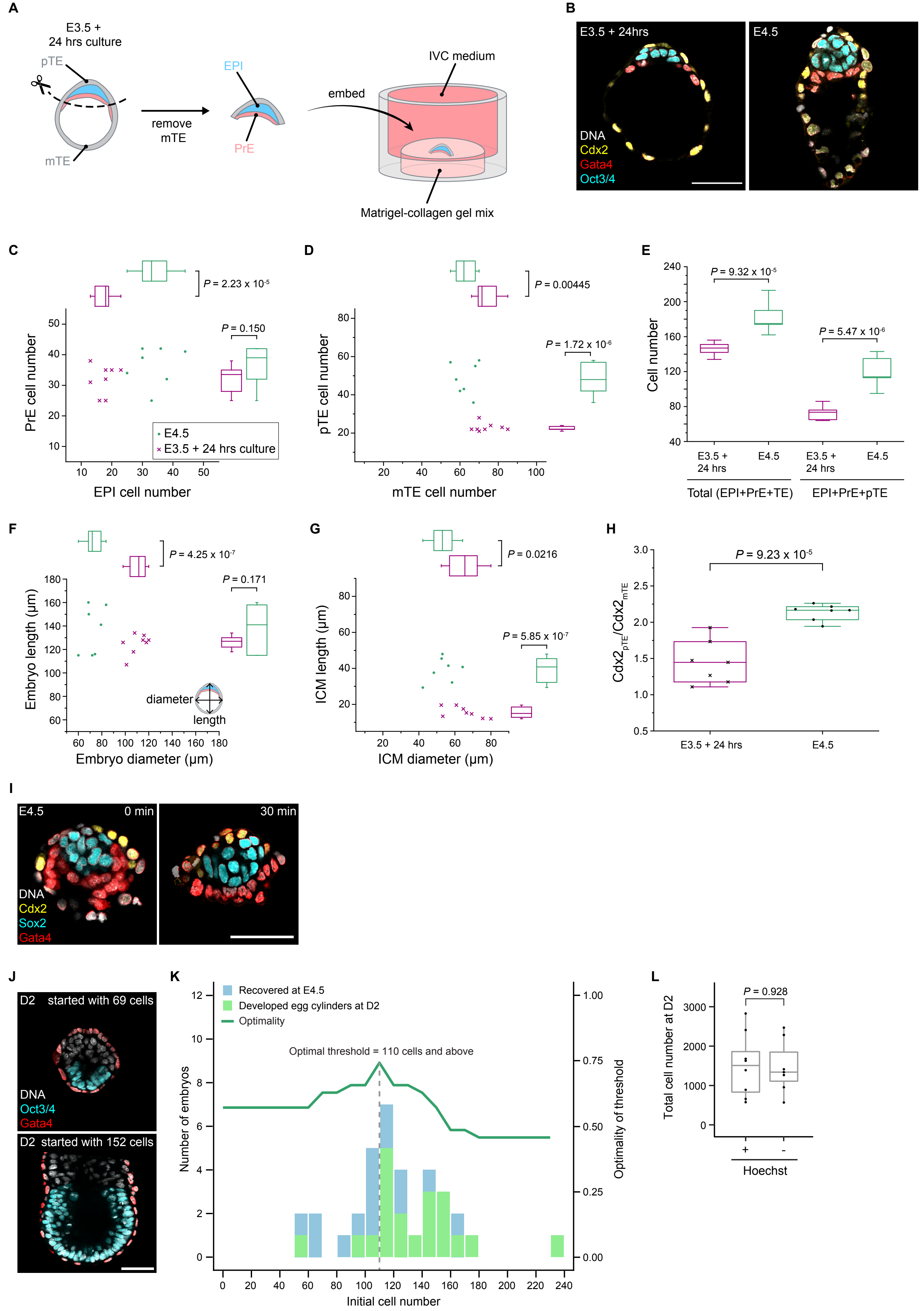
A
E3.5 +
24 hrs culture
pTE
mTE
remove
mTE
EPI
PrE
embed
IVC medium
Matrigel-collagen gel mix
B
E3.5 + 24hrs
E4.5
DNA
Cdx2
Gata4
Oct3/4
C
P = 2.23 x 10-5
P = 0.150
PrE cell number
EPI cell number
E4.5
E3.5 + 24 hrs culture
D
P = 0.00445
P = 1.72 x 10-6
pTE cell number
mTE cell number
E
P = 9.32 x 10-5
P = 5.47 x 10-6
Cell number
E3.5 + 24 hrs
E4.5
Total (EPI+PrE+TE)
EPI+PrE+pTE
F
P = 4.25 x 10-7
P = 0.171
Embryo length (µm)
Embryo diameter (µm)
diameter
length
G
P = 0.0216
P = 5.85 x 10-7
ICM length (µm)
ICM diameter (µm)
H
P = 9.23 x 10-5
Cdx2pTE/Cdx2mTE
E3.5 + 24 hrs
E4.5
I
E4.5
0 min
30 min
DNA
Cdx2
Sox2
Gata4
J
D2 started with 69 cells
DNA
Oct3/4
Gata4
D2 started with 152 cells
K
Recovered at E4.5
Developed egg cylinders at D2
Optimality
Optimal threshold = 110 cells and above
Number of embryos
Optimality of threshold
Initial cell number
L
P = 0.928
Total cell number at D2
Hoechst

Figure S2. Quality control for E4.5 mouse blastocysts suitable for 3D-geec, Related to Figure 2, 3 and 4

(A) Schematic of the experimental workflow. Embryos are recovered at E3.5 and cultured in medium for 24 hours, followed by microsurgery to remove mTE. Then embryos are embedded in Matrigel-collagen mix and submerged in IVC medium.

(B) Immunofluorescence images of a representative embryo at E3.5 followed by 24 hours culture and E4.5, stained for Oct3/4$^+$ EPI, Gata4$^+$ PrE, Cdx2$^+$ TE and DNA. *n* = 25 (E3.5 + 24hrs) and 28 (E4.5) embryos.

(C-H) Scatter plots with boxplots showing the cell number of EPI, PrE, mTE and pTE (C-E), dimensions of embryo (F) and ICM (G), and ratio of Cdx2 expression in pTE to mTE (H) in embryos shown in (B). *n* = 8 (E3.5 + 24hrs) and 7 (E4.5) embryos.

(I) Immunofluorescence of representative E4.5 blastocysts upon mechanical dissection of mTE (left) and after 30 minutes incubation in IVC1 medium (right) stained for Sox2$^+$ EPI, Gata4$^+$ VE and Cdx2$^+$ TE. Note the tissue shape change, due possibly to pTE contraction.

(J) Immunofluorescence of 3D-geec embryos at D2 with initial cell number 69 (top) and 152 (bottom) stained for Oct3/4$^+$ EPI and Gata4$^+$ VE.

(K) Histograms of the number of embryos recovered at E4.5 (blue) and those developed to egg cylinder by 3D-geec at D2 (green) for a given total number of ICM and pTE cells (bin size = 10), with a line chart of optimality (see STAR Methods). A threshold level of ≥110 cells provides the best optimality of 0.74. *n* = 35 embryos; *N* = 4.

(L) The total EPI and VE cell number of Hoechst-treated and -untreated 3D-geec embryos at D2. Hoechst-treated: *n* = 8 embryos, Hoechst-untreated: *n* = 7 embryos; *N* = 2.

P values calculated using *t*-test (C-H) and Mann-Whitney *U* test (L).

Scale bars, 50 μm.

Figure S3

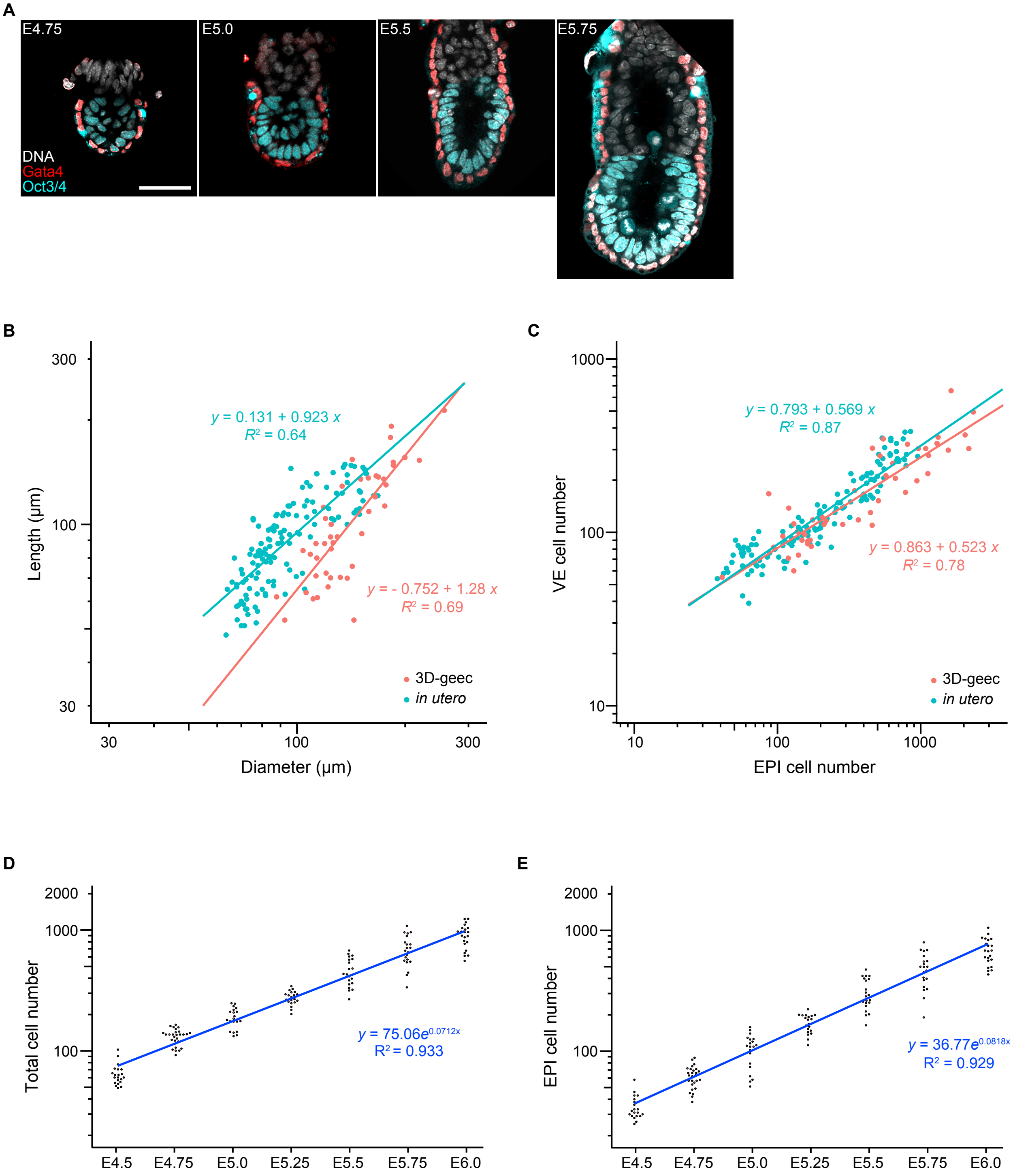
A
E4.75
E5.0
E5.5
E5.75
DNA
Gata4
Oct3/4
B
Length (µm)
Diameter (µm)
y = 0.131 + 0.923 x
R² = 0.64
y = - 0.752 + 1.28 x
R² = 0.69
3D-geec
in utero
C
VE cell number
EPI cell number
y = 0.793 + 0.569 x
R² = 0.87
y = 0.863 + 0.523 x
R² = 0.78
3D-geec
in utero
D
Total cell number
y = 75.06e^0.0712x
R² = 0.933
E4.5
E4.75
E5.0
E5.25
E5.5
E5.75
E6.0
E
EPI cell number
y = 36.77e^0.0818x
R² = 0.929
E4.5
E4.75
E5.0
E5.25
E5.5
E5.75
E6.0

Figure S3. Quantitative characterization of peri-implantation mouse embryos, Related to Figure 3

(A) Immunofluorescence of a representative embryo developed *in utero* at E4.75, E5.0, E5.5 and E5.75 stained for Oct3/4$^+$ EPI and Gata4$^+$ VE.

(B, C) The trends of 3D-geec and *in utero* embryos shown in Figure 3B and 3C in terms of dimensions (B, P = 0.0076) and cell numbers (C, P = 0.2451). P values calculated using two-way ANOVA.

(D, E) Plots showing the total (EPI and PrE/VE) cell number (D) or EPI cell number (E) against the developmental stage of the recovered embryo defined by the time of recovery. Based on the cell numbers and the regression lines, embryos can be re-defined as a quantitatively normalized stage (nE; see STAR Methods). Equation of regression line for total cell number is $y = 75.061e^{0.0712x}$; that for EPI cell number is $y = 36.77e^{0.0818x}$. n = 21 (E4.5), 28 (E4.75), 20 (E5.0), 20 (E5.25), 21 (E5.5), 21 (E5.75) and 22 (E6.0) embryos.

Scale bar, 50 μm.

Figure S4

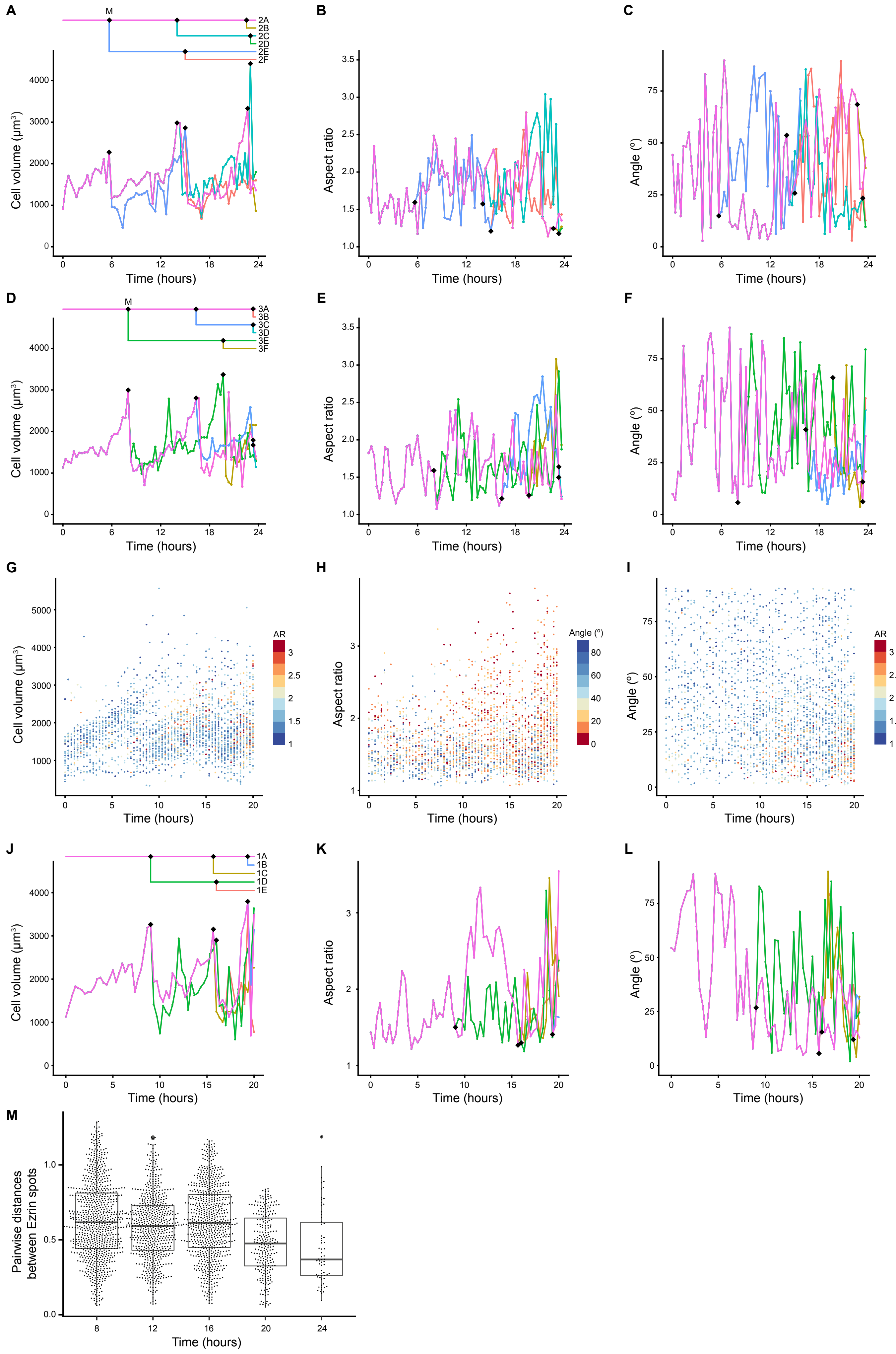

A
B
C
D
E
F
G
H
I
J
K
L
M
Cell volume (μm³)
Aspect ratio
Angle (°)
Time (hours)
Pairwise distances between Ezrin spots
AR
Angle (°)
M
2A
2B
2C
2D
2E
2F
3A
3B
3C
3D
3E
3F
1A
1B
1C
1D
1E

Figure S4. Quantitative analysis of cellular dynamics in peri-implantation development, Related to Figure 6

(A-F) Cell volume (A and D), aspect ratio (B and E) and long-axis radial alignment against the outer embryonic surface (C and F) of two additional representative lineages in the embryo shown in Figure 6B. *n* = 18 lineages. Black diamonds in a lineage tree and in plots represent mitoses.

(G-I) Cell volume (G), aspect ratio (H) and long-axis radial alignment against the outer embryonic surface (I) of EPI cells in another embryo. Cells are analyzed every 20 min for 20 hours of 3D-geec until pro-amniotic cavity formation.

(J-L) Cell volume (J), aspect ratio (K) and long-axis radial alignment against the outer embryonic surface (L) of a representative lineage of EPI cells in the embryo (G-I).

(M) Pair-wise distance of Ezrin spot signals scaled to the diameter of the embryo shown in Figure 6L. Note that the number of pair-wise distance measurements decreases over 24 hours as Ezrin spots fused.

See also Videos S5 and S6.

Figure S5

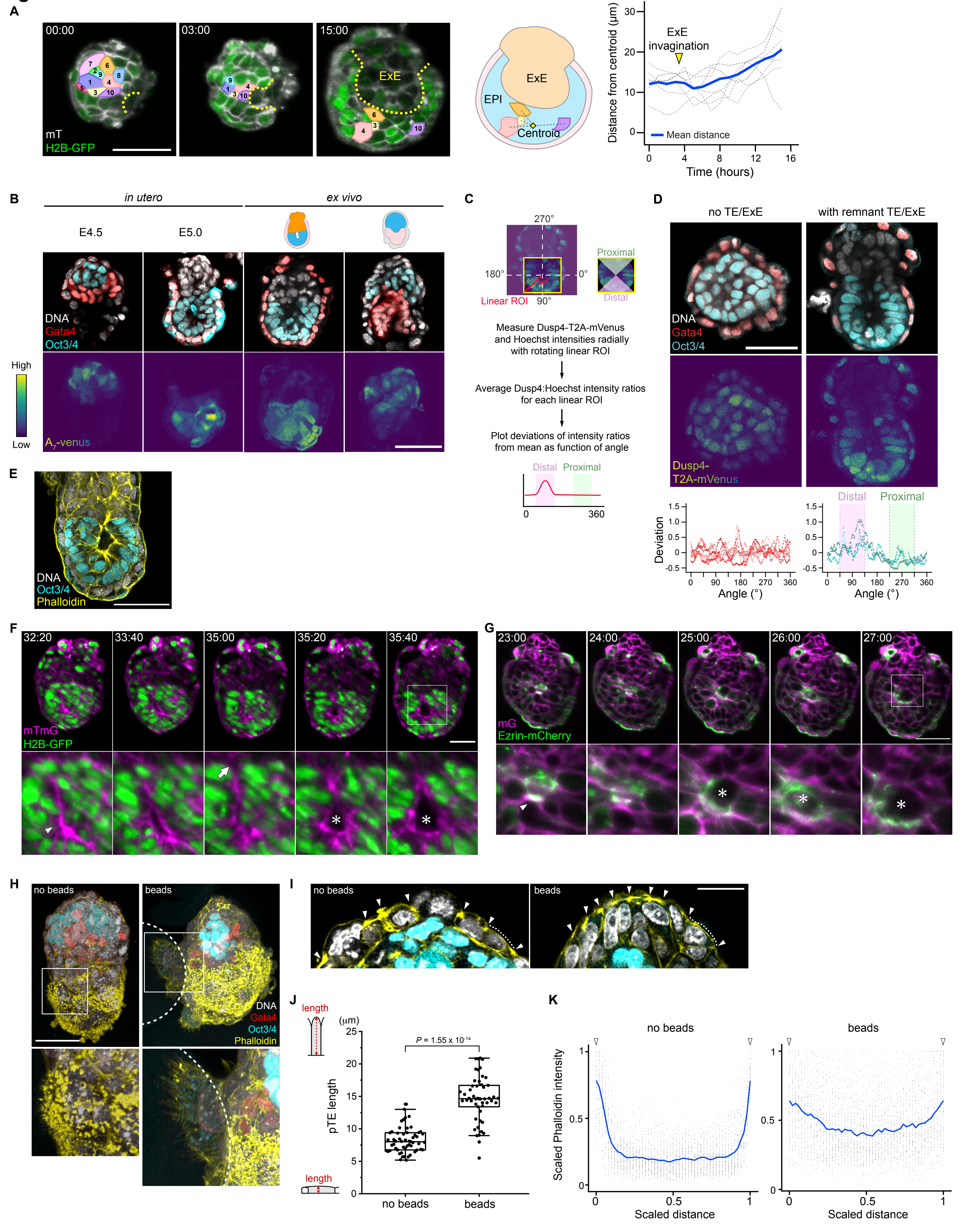
A
00:00
03:00
15:00
mT
H2B-GFP
ExE
EPI
Centroid
ExE invagination
Distance from centroid (µm)
Mean distance
Time (hours)
B
in utero
ex vivo
E4.5
E5.0
DNA
Gata4
Oct3/4
High
Low
A_7-venus
C
270°
180°
0°
90°
Linear ROI
Proximal
Distal
Measure Dusp4-T2A-mVenus and Hoechst intensities radially with rotating linear ROI
Average Dusp4:Hoechst intensity ratios for each linear ROI
Plot deviations of intensity ratios from mean as function of angle
D
no TE/ExE
with remnant TE/ExE
Dusp4-T2A-mVenus
Deviation
Angle (°)
E
Phalloidin
F
32:20
33:40
35:00
35:20
35:40
mTmG
H2B-GFP
G
23:00
24:00
25:00
26:00
27:00
mG
Ezrin-mCherry
H
no beads
beads
I
J
length
(µm)
$P = 1.55 \times 10^{-14}$
pTE length
K
Scaled Phalloidin intensity
Scaled distance

Figure S5. Impact of ExE on EPI cell dynamics and patterning, Related to Figure 7

(A) Tracking of 10 neighboring EPI cells in a representative H2B-GFP;mT embryo during the first 15 hours of 3D-geec. Mean distance from the centroid of tracked cells is used as a measure of cell dispersion. *t* = hours:minutes. Yellow broken lines mark the boundary between ExE and EPI.

(B) Immunofluorescence of representative A_7-Venus embryos developed *in utero*, or *ex vivo* in the presence and absence of ExE, stained for Oct3/4$^+$ EPI and Gata4$^+$ VE. *n* = 3 (E4.5), 8 (E5.0), 2 (*ex vivo*, with ExE) and 3 (*ex vivo*, without ExE) embryos.

(C) Schematic to quantify the spatial distribution of Dusp4 signals in EPI (see also STAR Methods).

(D) Representative images of embryos without TE (hence no ExE) (left) and with remnant TE (hence forming a small ExE) (right), immunostained for Oct3/4$^+$ EPI, Gata4$^+$ VE and DNA. Deviations of endogenous Dusp4-T2A-mVenus signal normalized by Hoechst was plotted as described in (C). *n* = 8 (no TE/ExE), 3 (with remnant TE/ExE) embryos.

(E) Immunofluorescence of a representative E5.0 embryo developed *in utero*, stained for actin and Oct3/4$^+$ EPI.

(F, G) Time-lapse images of representative H2B-GFP;mT (F) and mG;Ezrin-mCherry (G) mouse embryos developed *ex vivo* in the presence of ExE from E4.5 (*t* = 00:00, hours:minutes). Arrowheads and asterisks mark rosettes and the pro-amniotic cavity, respectively. An arrow indicates a fissure-like structure connecting to the boundary between EPI and ExE.

(H) Representative 3D projection images of E4.5 embryos embedded in Matrigel-collagen mix, cultured for 18 hours in the absence (left) or presence (right) of adhesive beads in contact (white broken lines), and immunostained for Oct3/4$^+$ EPI, Gata4$^+$ PrE, actin and DNA. Enlarged views of the insets (white boxes) showing mTE cells.

(I) Enlarged 2D views of pTE cells in embryos shown in (H). Arrowheads indicate the cell-cell junctions.

(J) The length of pTE cells in embryos shown in (H). *n* = 55 cells from 11 embryos (no beads), 50 cells from 10 embryos (beads attached to the lateral side of blastocysts).

(K) Subcellular localization of actin along the apical surface of pTE cells in embryos shown in (H). Scaled intensity profiles obtained by tracing a line along the apical membrane of a pTE cell (white broken lines in (I)). Arrowheads indicate the cell-cell junctions. Blue lines presented as mean. *n* = 54 cells (no beads), 56 cells (beads attached to the lateral side of blastocysts).

P value calculated using Mann-Whitney *U* test.

Scale bars, 50 µm (A-H), 20 µm (I).

Supplemental Tables

Mouse Line	Primer ID	Primer Sequence	PCR Product Size, bp
mTmG and mG	oIMR7318	CTCTGCTGCCTCCTGGCTTCT	WT allele, 330; Knock-in allele, 250
	oIMR7319	CGAGGCGGATCACAAGCAATA	
	oIMR7320	TCAATGGGCGGGGGTCGTT	
H2B-GFP	CAG-Fw	GGCTTCTGGCGTGTGACCGGC	Tg allele, 900
	EXFP-Rv	GTCTTGTAGTTGCCGTCGTC	
GFP-Myh9	GFP-Myh9_1	CTGTCACATGGCTCATGTTC	WT allele, 400; Knock-in allele, 200
	GFP-Myh9_2	GCCGGACACGCTGAACTTGT	
	GFP-Myh9_3	GCCCTGAGTAGTATCGCTCC	
Cdx2-GFP	Cdx2-Fw	ATGGTTCCGTTCCCTGGTTC	WT allele, 1400; Knock-in allele, 750
	GFP-Rv	GCGGACTTGAAGAAGTCGTGCTGCTT	
	Cdx2-EX3	AGGCTTGTTTGGCTCGTTACAC	
Lefty1-mVenus	lefty1-53	CAGGCATCAAGCAGAGAACG	WT allele, 324; Tg allele, 880
	oIMR1416	TCCTTGAAGAAGATGGTGCG	
	oIMR0042	CTAGGCCACAGAATTGAAAGATCT	
	oIMR0043	GTAGGTGGAAATTCTAGCATCATCC	
A_7-Venus	Venus-Fw	ACGTAAACGGCCACAAGTTC	Tg allele, 400
	Venus-Rv	TGTCGGCGGTGATATAGACG	
Ezrin-mCherry	Ezrin-mCh-Fw	TGGCCATCATCAAGGAGTTCATG	Tg allele, 348
	Ezrin-mCh-Rv	TGTAGATGAACTCGCCGTCCTG	
Dusp4-T2A-3xmVenus	Dusp4-wt-Fw	GCCCGCCAAGGTCCCTAATC	WT allele, 464; Knock-in allele, 639
	Dusp4-wt-Rv	GGGGGCCGACTCTGGATTTG	
	mVenus-Rv	ACGCTGAACTTGTGGCCGTT	

Table S1. Genotyping primers and PCR product sizes, Related to STAR Methods.
